# A Case of Strangulated Femoral Hernia

**Published:** 1918-06-15

**Authors:** Albert Wm. Gregorson

**Affiliations:** (Acting) Medical Superintendent, Edmonton Infirmary.


### A CASE OF STRANGULATED FEMORAL HERNIA.

By ALBERT WM. GREGORSON, M.D., Ch.B. Glasgow, F.R.F.P.S., (Acting) Medical Superintendent, Edmonton Infirmary.

The following case, presenting somewhat unusual features, recently came under my care. E. B., aged eighty-three, widow, was admitted to Edmonton Infirmary on December 20, 1916, for the relief of "strangulated femoral hernia." Patient stated "she had always been able to put it back, but had not been able to do so for two days, and had been vomiting and very ill"; further, "that she was going to die, as her sister had died from the same complaint, but was quite willing to have anything done that might save her life."

On admission there was a mass the size of an orange in the right groin. This had been present for thirty years. The face was pale, the pulse feeble, the tongue furred, and the abdomen distended. Fæcal vomiting was present, and the patient appeared to be dying. Temperature, 98.2 F.; pulse, 90; respirations, 24 per minute. The urine, which was acid, had a specific gravity of 1026. It contained no albumin, blood, or sugar.

### Operation.

The patient was anæsthetised with chloroform, followed by a little ether. A vertical incision was made over the swelling. The sac was found to be tough and very vascular, being covered with a network of dilated veins. By careful dissection the sac was separated and opened, and there presented a knuckle of bowel which was quite black and lustreless. At this stage of the operation the black part of the bowel perforated. The point of perforation was secured by a clamp and a fine silk ligature was passed round it. A double suture was also applied for safety, the gangrenous area was then buried by bringing the two lateral folds of the sero-muscle wall over it with a continuous suture, the gangrenous patch being thus completely invaginated. This proceeding was possible as the segment of the intestine was short, flexible, and not too friable. The other parts of the intestine seemed fairly healthy. These manipulations were carried out with the utmost care and gentleness, so as to avoid producing a further rupture or perforation. After swabbing with hot sterile water, the prolapsed gut was returned to the peritoneal cavity. The sac was twisted and placed as a pad to provide a covering for the orifice of the femoral ring. The wound was closed without drainage.

### After-Treatment.

One-sixtieth grain strychnine sulphate was given four hourly, and warmth was carefully maintained by hot-water bottles. Three days after the operation a mixture containing sodium sulphate, magnesium sulphate, and tincture nucis vomicæ was given, until a satisfactory movement of the bowels was obtained. Twelve days after the operation the patient had an attack of acute bronchitis, which threatened her life, but under suitable treatment recovery was brought about, the attack lasting ten days. The wound healed by first intention, and convalescence proceeded uninterruptedly. The patient was discharged from the infirmary on March 22, 1917, perfectly well.

Prior to the operation the patient had been working regularly as a tram cleaner, and when the hope of regaining health came home to her she was anxious to resume this work as soon as possible, so as to earn her living. The admirable courage, energy, and spirit of independence shown by this woman, eighty-three years of age, in her ardour to leave hospital so soon after an operation for the relief of a strangulated femoral hernia in order to resume the arduous duties of a tram cleaner and so earn her living are features which for themselves alone render her case worthy of publication.

[I beg to express indebtedness to Colonel Mort, R.A.M.C.—A. W. G.]

